# Stressful life events and non-suicidal self-injury among Chinese adolescents: A moderated mediation model of depression and resilience

**DOI:** 10.3389/fpubh.2022.944726

**Published:** 2022-08-04

**Authors:** Chang Wei, Zhiyong Li, Tao Ma, Xiaxia Jiang, Chengfu Yu, Qian Xu

**Affiliations:** ^1^Research Center for Rural Educational and Cultural Development of Key Research Base of Humanities and Social Sciences in Hubei Province, School of Education, Hubei University of Science and Technology, Xianning, China; ^2^School of Psychology, South China Normal University, Guangzhou, China; ^3^College of Education Science, Hubei Normal University, Huangshi, China; ^4^School of Psychology, Central China Normal University, Wuhan, China; ^5^School of Education, Guangzhou University, Guangzhou, China

**Keywords:** adolescents, stressful life events, resilience, depression, non-suicidal self-injury

## Abstract

Stressful life events are associated with an increased risk of non-suicidal self-injury (NSSI) in adolescence, but the mechanisms explaining this association are unclear. Based on the experiential avoidance model of NSSI, and the protective factor model of resilience, the current study tested depression as a mediator and resilience as a moderator of this association. Chinese adolescents (*N* = 643; *M*_*age*_ = 15.91; 52.10 % female), anonymously completed self-report measures in classrooms. Results showed that stressful life events was linked to adolescent NSSI in part because of adolescent depression, and resilience was a protective factor that buffered this effect. These findings can inspire practitioners to pay attention to the interaction of risk factors and protective factors when providing prevention and intervention for adolescent NSSI.

## Introduction

Non-suicidal self-injury (NSSI) is a perplexing behavior problem. It involves deliberately and directly destroying one's body tissue without suicidal intention, most commonly by cutting or carving oneself (1). Much of the research on NSSI has focused on adolescents, who show a higher rate of NSSI than other age groups (2). The prevalence of adolescent NSSI in China is estimated to be from 15.0 to 41.5% in community samples (3–6). The largest of these studies, with a sample of 18,900 Chinese junior and senior high school students, found that the prevalence of NSSI was 28.5% (5).

NSSI is known to be associated with adolescents' mental health problems (7, 8). In addition, although people do not engage in NSSI with suicidal intent, the behavior is closely related to suicidal behavior (9). Longitudinal results have shown that NSSI is a predictor of adolescents' suicidal behavior, not only a simple correlate (10). The high prevalence and the clinical significance of NSSI in adolescents motivate us to identify risk and protective factors that can inform effective prevention and intervention.

### Stressful life events and adolescent NSSI

Stressful life events refer to all kinds of negative events in individual life, which can have a negative impact on individuals' physical and mental health (such as family conflicts, classmate disputes, economic distress, death of relatives, and failure in examination) (11, 12). Previous research showed that stressful life events are strongly correlated with adolescents' risky behaviors (13, 14), consistent with general strain theory (15). In line with this theory, stressful life events have been shown to be associated with adolescent NSSI in correlational studies (16–18) and longitudinal studies (19–21). For example, in a longitudinal study, Baetens et al. (19) found that adolescents' stressful life events were significantly positively associated with NSSI 18 months later.

These studies document a direct link between stressful life events and adolescent NSSI. However, the underlying mechanism of this association, and the factors that may mitigate the risk, remain largely unexplored. Based on the experiential avoidance model of NSSI (22) and the protective factor model of resilience (23), the current research tested depression as a mediator, and resilience as a moderator, of the relationship between stressful life events and adolescent NSSI.

### Depression as a potential mediator

Depression is an emotional disorder that causes a persistent feeling of sadness and loss of interest, which is thought be caused in part by an inability to cope with stressful events (24). The first part of the mediation pathway in our model is the association between stressful life events and depression. The experiential avoidance model of NSSI (22) maintains that adolescents who experience stressful life events are more likely to have negative emotions such as depression, which in turn lead to NSSI. There is empirical evidence that stressful life events could increase the risk of adolescent depression (17, 52). In one longitudinal study of 1,094 Chinese adolescents, Chen et al. (52) found that peer victimization (a stressful life event) positively predicted depression six months later.

The second part of the mediation pathway that we tested was the association between adolescent depression and NSSI. It is generally assumed that NSSI serves the function of distracting the individual from emotional pain, an idea expressed by the experiential avoidance model of NSSI (22). Two longitudinal studies have shown a link between depression and later NSSI among adolescents (25, 53). For example, Wu et al. (25) found that depressive symptoms were correlated with NSSI one year later in a sample of 813 Chinese adolescents. Previous research also demonstrated that negative emotions could mediate the association between stressful life events and NSSI. For example, in a longitudinal study, Zhu et al. (6) found that anxiety symptoms mediated the relationship between cybervictimization and NSSI in a sample of 1987 adolescents in Chinese. Similarly, in a sample of 2464 Italian adolescents, Cipriano et al. (16) found that anger expression mediated the relationship between parental rejection and direct and indirect forms of NSSI.

Thus, based on theory and empirical research, we assert that depression is an important intermediary factor in the process by which stressful life events increase the risk of adolescent NSSI.

### Resilience as a moderator

Not all adolescents who experience stressful life events, and not all adolescents who experience depression, engage in NSSI. This suggests that there may be factors that lower the risk of NSSI even under these adverse conditions. One of these factors could be the adolescent's resilience. Resilience refers to an individual's ability to positively adapt to the environment even through adversity, including two critical conditions: exposure to significant adversity (such as exposure to community violence, parent mental illness, and poverty) and positive adaptation (good academic performance, positive relationships with teachers or classmate) (26, 27). According to the protective factor model of resilience (23), resilience can reduce the effects of environmental risk on negative outcomes. In our study, these ideas correspond to the environmental risk due to stressful life events and the negative outcomes of depression and NSSI.

There is also evidence that resilience could be an important influence on the mediation process of interest in the current study. There are several studies that can inform our hypotheses. Wang and Liu (28) found that resilience weakened the impact of stressful life events on delinquency in a sample of 306 Chinese adolescents whose parents had moved away in search of work. Ye et al. (29) found that resilience weakened the impact of peer victimization on depression in a sample of 721 Chinese children. Wu et al. (30) found that resilience buffered the association between depression and NSSI in a sample of 813 adolescents. In the current research, we examined whether resilience buffered the risk effects of stressful life events on adolescent NSSI.

### The present study

**Figure 1:**
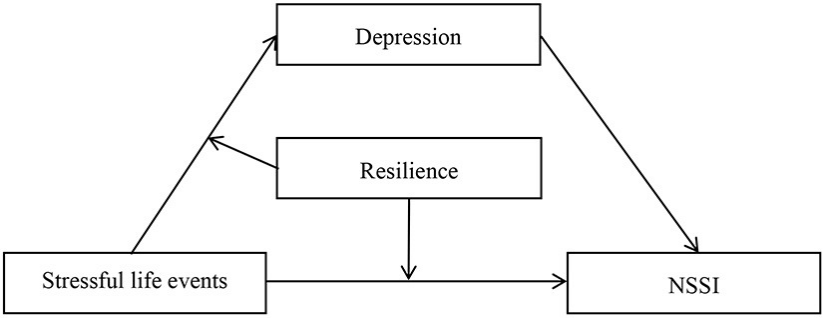
The proposed mediated moderation model. NSSI, non-suicidal self-injury.

On the basis of the experiential avoidance model of NSSI (22) and the protective factor model of resilience (23), this study tested a moderated mediation model in which stressful life events are a risk factor for adolescent NSSI *via* depression, and resilience is a protective factor against NSSI. The proposed model can be seen in Figure 1.

It should be noted that the current study was conducted during the COVID-19 pandemic. During this period several studies showed a negative impact of COVID-19 on adolescents' psychology (48, 49) and behavior (31–33). Thus, the stressful life events reported by the adolescents were occurring in the context of overarching, long term stress. In this respect their scores on the measure of stressful life events may be an underestimate of their true stress. Scores on the other study variables might also be affected by life during the pandemic; depression and NSSI might be higher, and resilience might be lower. This is an unfortunate but unique opportunity to study this group of adolescents, who can be considered high-risk because of their experiences related to COVID-19. Thus, this study exaned the prevalence rate of NSSI among adolescents during the COVID-19 epidemic.

We proposed the following hypotheses: (1) The prevalence rate of NSSI among adolescents will be higher during the COVID-19 epidemic than it was before the pandemic; (2) stressful life events will be positively associated with adolescent NSSI; (3) depression will mediate the association between stressful life events and NSSI; (4) resilience will weaken the direct and indirect relations between stressful life events and adolescent NSSI. Specifically, resilience will buffer the direct effect of stressful life events on NSSI, and will buffer the association between stressful life events and depression.

## Methods

### Participants

A random cluster sampling method was used to recruit students from a middle school in Hubei Province, in central China. A total of 643 students participated in our study. No adolescents had obvious mental or physical illness. The average age was 15.91 years old (*SD* = 0.74; age range: 14–17 years). There were 335 girls (52.1%) and 308 boys (47.9%), and 78.5 % had one or more sibling.

### Procedure

This research was approved by the Ethics Committee of Hubei University of Science and Technology. The data were collected from March to April 2022. Parents provided written informed consent and adolescents provided assent. The adolescents were told that they could withdraw from the study at any time without penalty. They were also told that their responses would be kept confidential. All the data collected were anonymous. The questionnaires were administered by the researchers of this study and trained graduate students under their supervision. All participants completed the self-report measures in their classrooms.

### Measures

#### Stressful life events

The number of stressful life events was measured with the Chinese Language Adolescent Life Events Scale (12). Participants were asked to report the seriousness of each listed event in the past year (such as “family conflicts,” and “classmate disputes”). There are 27 items on the scale, and each item is scored on a 6-point scale (0 = none to 5 = very serious). Mean scores were used for analysis, with higher scores indicating higher severity of stressful life events. Cronbach's alpha in this study was 0.93.

#### Depression

Depression was measured with the Chinese version (34) of the Center for Epidemiological Studies-Depression Scale (35). Participants were asked to assess how often they experienced depressive symptoms in the past week (such as “I feel depressed,” “I feel lonely,” and “It's hard for me to concentrate”). There are 20 items on the scale, and each item is rated on a 4-point scale (0 = <1 to 3 = 5–7 days). Mean scores were used for analysis, with a higher score indicating a higher level of depression. Cronbach's alpha in this study was 0.85.

#### Resilience

Resilience was measured with the Resilience Scale for Chinese Adolescents (36). This questionnaire includes 27 items (such as “After experiencing setbacks, I generally become more mature and experienced”) covering five dimensions: goal focus, interpersonal assistance, family support, emotional control, and positive cognition. Each item is rated on a 5-point scale (1 = almost always untrue to 5 = almost always true). Mean scores were used for analysis, with higher scores indicating a higher level of resilience. Cronbach's alpha in this study was 0.76.

#### NSSI

We assessed twelve NSSI behaviors (37) selected from the Deliberate Self-Harm Inventory (38), such as cutting, carving, burning, and severely scratching oneself. These 12 items were chosen because they are common forms of NSSI among adolescents (1), and have been shown to have good psychometric properties in previous studies of Chinese adolescents (25, 30, 37, 39). Participants were asked to report how often they had engaged in NSSI in the past year. Each item is rated on a 6-point scale (0 = never to 5 = 5 or more times). The 12 item scores were added, with higher scores indicating a higher frequency of NSSI. Cronbach's alpha in this study was 0.91.

#### Control variables

There are significant gender differences in NSSI among adolescents, with girls being more at risk than boys (40, 41). NSSI is also associated with age (1, 2). The students all came from neighborhoods near the school, and so they had a similar socioeconomic status. Therefore, the analyses controlled for age and gender (0 = female; 1 = male).

### Statistical analyses

We used SPSS 21.0 to generate descriptive statistics and correlations. Mediation and moderation effects were calculated in Mplus 8.3. Missing data were handled *via* the full information maximum-likelihood (FIML) estimation method. We used bootstrapping with 2000 iterations to test the significance of each direct and indirect path. By convention, the model fit is considered good when χ^2^/d*f* < 5, CFI >0.90, TLI >0.90, RMSEA < 0.08, and SRMR < 0.08 (42).

## Results

### Preliminary analyses

The results showed that 237 of the 643 adolescents endorsed one or more NSSI behaviors in the past 12 months, an estimated 12 month prevalence of 36.9%. The means, standard deviations, and correlation coefficients for all research variables are displayed in Table 1. Stressful life event scores were positively correlated with depression (*r* = 0.43, *p* < 0.001) and NSSI (*r* = 0.22, *p* < 0.001); depression was also positively correlated with NSSI (*r* = 0.31, *p* < 0.001). Resilience was negatively correlated with stressful life events (*r* = −0.25 *p* < 0.001), depression (*r* = −0.39, *p* < 0.001) and NSSI (*r* = −0.15, *p* < 0.001).

**Table 1:** Descriptive statistics and correlations for all variables.

**Variable**	**1**	**2**	**3**	**4**	**5**	**6**
1. Gender	1.00					
2. Age	0.07	1.00				
3. SLE	0.03	0.05	1.00			
4. Resilience	−0.02	0.05	−0.25^***^	1.00		
5. Depression	−0.08^*^	0.04	0.43^***^	−0.39^***^	1.00	
6. NSSI	0.04	0.05	0.22^***^	−0.15^***^	0.31^***^	1.00
*Mean*	0.48	15.91	1.27	3.23	0.73	1.33
*SD*	0.50	0.74	0.75	0.39	0.43	3.54

* p < 0.05,

*** p < 0.001.

Gender was dummy coded as 1 = male, 0 = female. SLE, stressful life events; NSSI, non-suicidal self-injury.

### Mediation effect of depression

**Figure 2:**
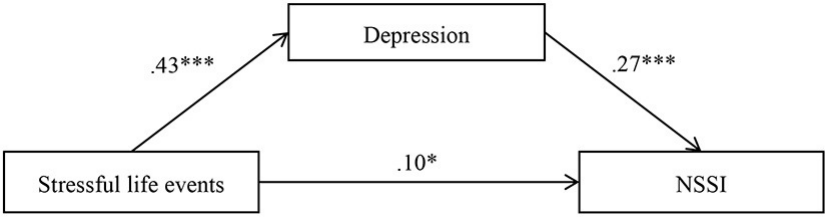
Model of the mediating role of depression between stressful life events and NSSI. NSSI, non-suicidal self-injury. The numbers are standardized regression coefficients. Path coefficients between control variables (age and gender) and each of the variables in the model are not displayed. Of the paths involving control variables, gender (dummy coded as 1 = male, 0 = female) was significantly related to depression (*b* = −0.10, *SE* = 0.04, *p* < 0.01). *p < 0.05. ***p < 0.001.

The hypothesized mediation model showed a good fit to the data, χ^2^/d*f* = 2.23, CFI = 0.99, TLI = 0.96, RMSEA = 0.04, and SRMR = 0.02. Figure 2 displays the results for each path in the proposed model. Stressful life events positively predicted depression (*b* = 0.43, *SE* = 0.03, *p* < 0.001) and NSSI (*b* = 0.10, *SE* = 0.04, *p* < 0.05), and depression positively predicted NSSI (*b* = 0.27, *SE* = 0.05, *p* < 0.001). Bootstrapping analyses showed that depression partially mediated the pathway from stressful life events to NSSI (indirect effect = 0.12, *SE* = 0.03, 95% CI = [0.07, 0.17]).

### Moderated mediation

**Figure 3:**
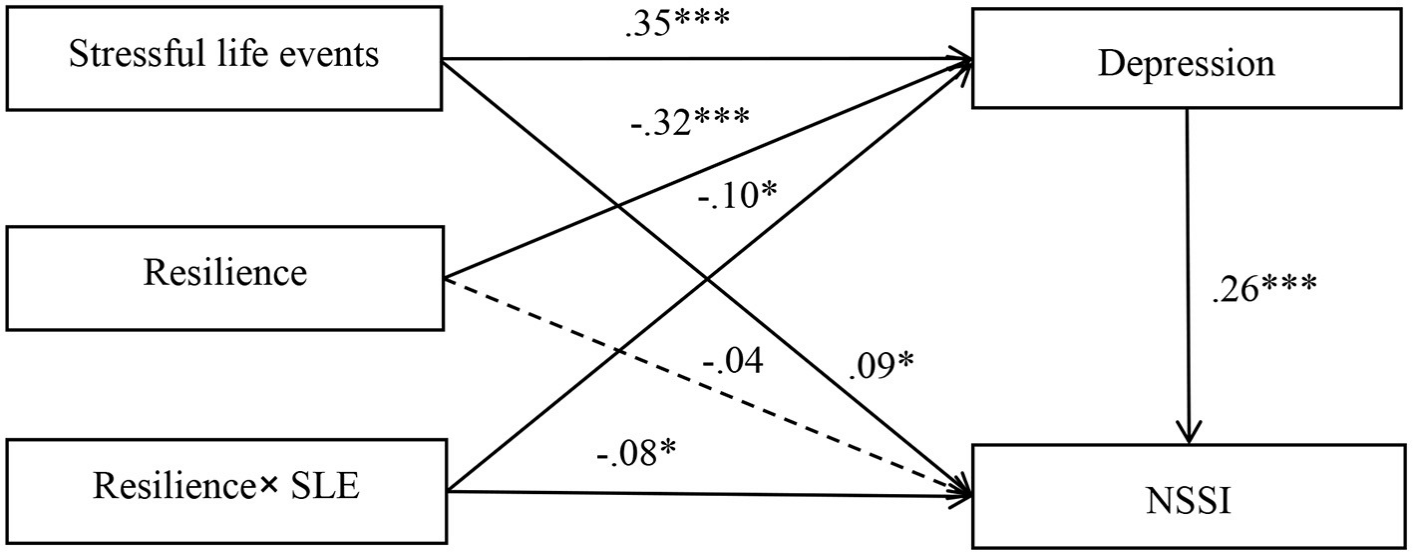
Model of the moderating role of resilience on the direct and indirect relationship between stressful life events and NSSI. NSSI, non-suicidal self-injury. The numbers are standardized regression coefficients. Path coefficients between control variables (gender and age) and each of the variables in the model are not displayed. Of the paths involving control variables, gender (dummy coded as 1 = male, 0 = female) was significantly associated with depression (*b* = −0.11, *SE* = 0.03, *p* < 0.01). *p < 0.05. ***p < 0.001.

The moderated mediation model represented in Figure 3 revealed a good fit to the data: χ^2^/d*f* = 1.36, CFI = 0.99, TLI = 0.99, RMSEA = 0.02, and SRMR = 0.02. Stressful life events (*b* = 0.09, *SE* = 0.04, *p* < 0.05) and depression (*b* = 0.26, *SE* = 0.06, *p* < 0.001) were significantly associated with NSSI. Stressful life events (*b* = 0.35, *SE* = 0.03, *p* < 0.001) and resilience (*b* = −0.32, *SE* = 0.04, *p* < 0.001) were significantly associated with depression. More importantly, resilience significantly moderated the impact of stressful life events on depression (*b* = −0.10, *SE* = 0.04, *p* < 0.05) and NSSI (*b* = −0.08, *SE* = 0.03, *p* < 0.05).

**Figure 4:**
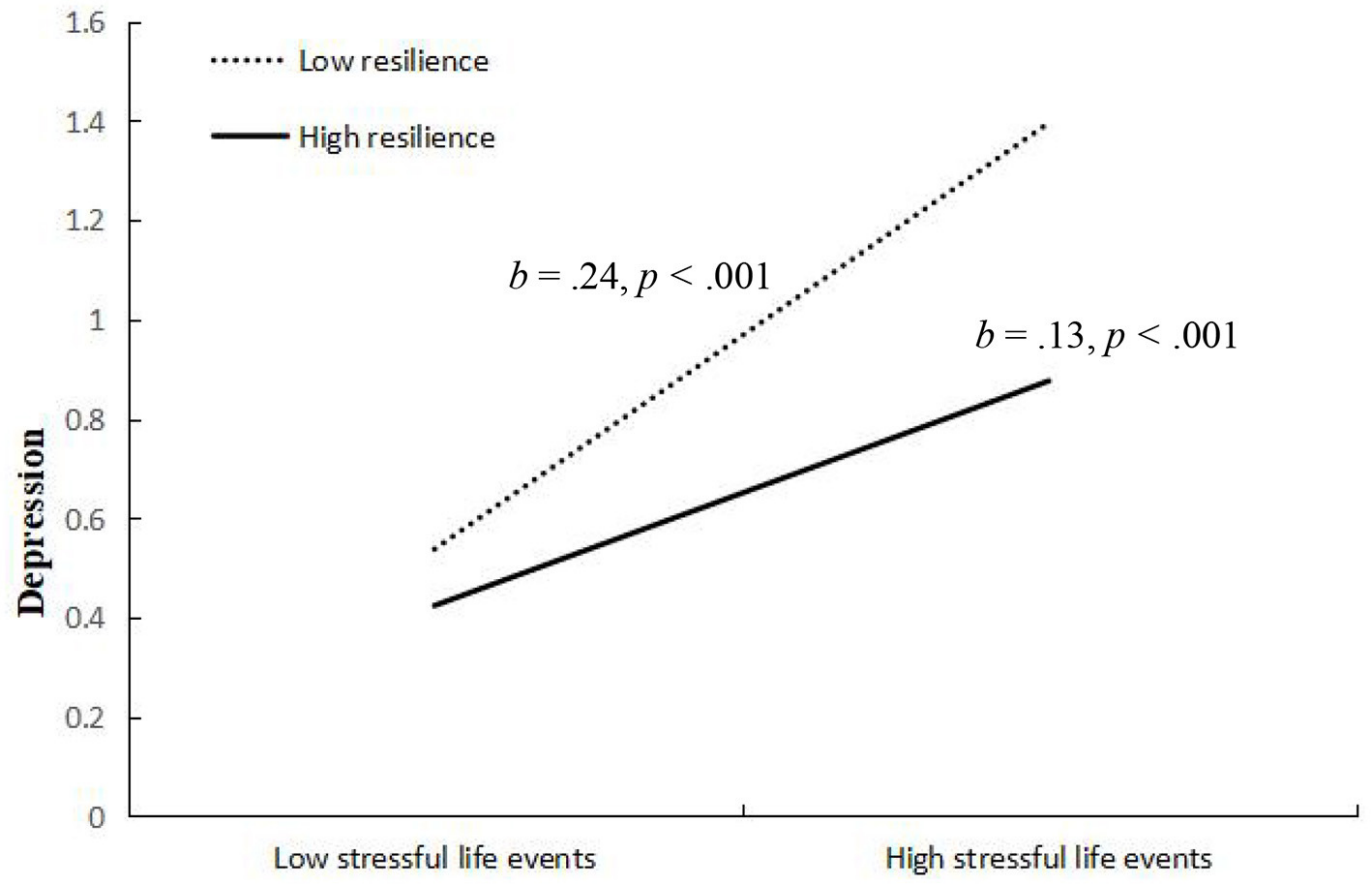
Interactive effect of stressful life events and resilience on depression. Resilience is graphed for two groups of participants: high resilience (1 SD above the mean) and low resilience (1 SD below the mean).

**Figure 5:**
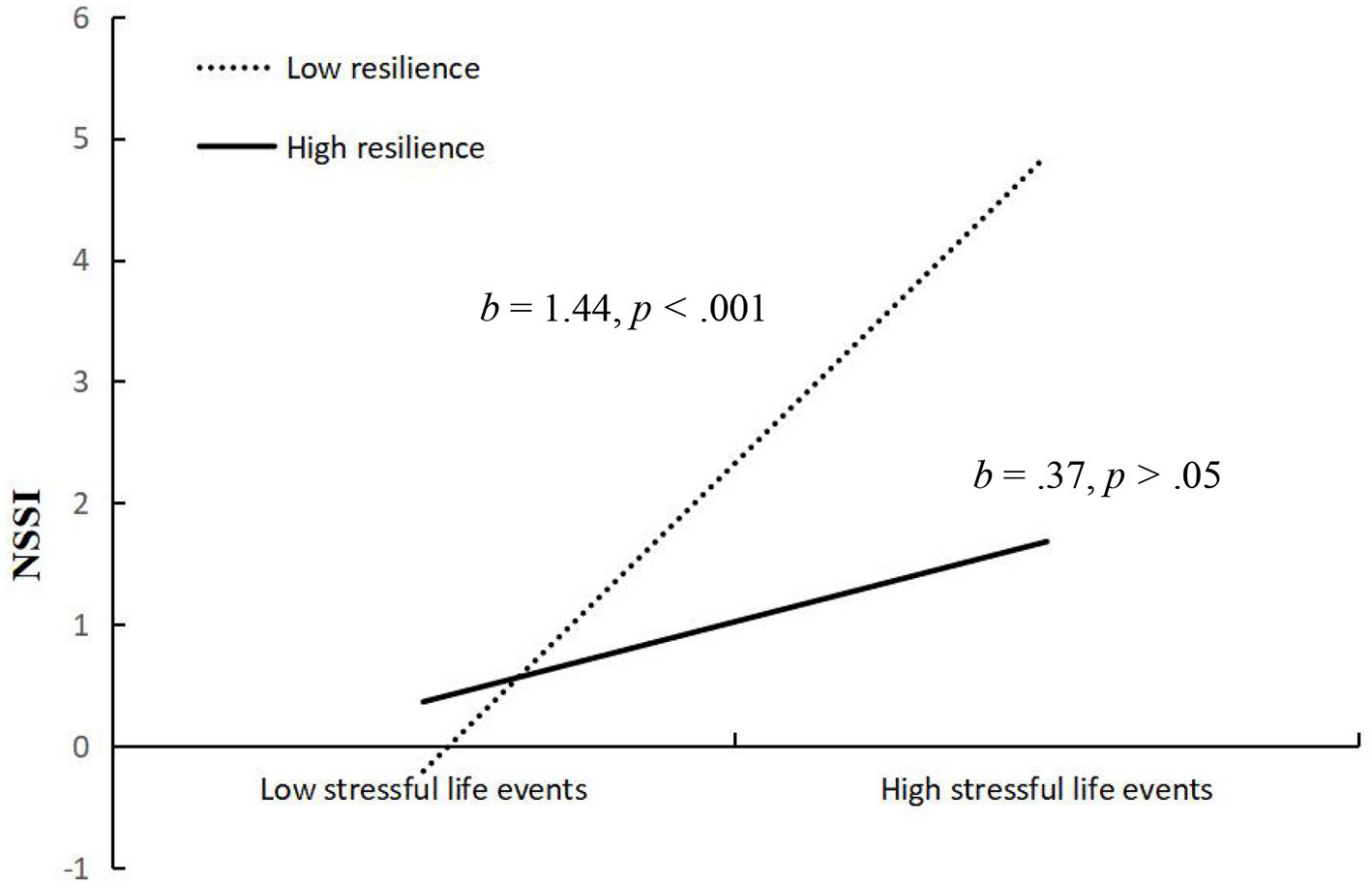
Interactive effect of stressful life events and resilience on NSSI. NSSI, non-suicidal self-injury. Resilience is graphed for two groups of participants: high resilience (1 SD above the mean) and low resilience (1 SD below the mean).

We conducted simple slopes tests to better understand the results regarding resilience as a moderator. As depicted in Figure 4, the relationship between stressful life events and depression was significant at both high (at 1 SD above the mean) and low (at 1 SD below the mean) levels of resilience, but the association was weaker when resilience was high. To be specific, when youth showed lower resilience, the relation between stressful life events and depression was significant (*b* = 0.24, *SE* = 0.03, *p* < 0.001). However, when youth showed higher resilience, this relation was weaker, although still statistically significant (*b* = 0.13, *SE* = 0.03, *p* < 0.001). Figure 5 shows the relationship between stressful life events and NSSI at low (at 1 SD below the mean) and high (at 2 SD above the mean) levels of resilience. When youth showed lower resilience, the relation between stressful life events and NSSI was significant (*b* = 1.44, *SE* = 0.29, *p* < 0.001). However, when youth showed higher resilience, this relation was non-significant (*b* = 0.37, *SE* = 0.32, *p* >0.05). Namely, resilience weakened the effects of stressful life events on depression and NSSI.

## Discussion

The results showed that the prevalence of adolescent NSSI in our sample was 36.9%, which is similar to the rates of 32% (43) and 40.9% (33) reported in other samples of adolescents during the COVID-19 pandemic. This is higher than the rate of 20.8% (37) reported in a sample of adolescents before the COVID-19 outbreak, in a study using the same NSSI measurement tool as in our study. Because of the high prevalence and clinical significance of these behaviors, the topic is worthy of more attention during the COVID-19 pandemic. In the current study we tested factors that increased and decreased the risk of NSSI in a sample of Chinese adolescents aged 14–17.

Although previous studies have demonstrated that depression could mediate the link between stressful life events and NSSI, resilience as an important moderator is the major strength of this work. Previous research has focused on just parts of the moderated mediation model we test in the current study. Wang and Liu (28) studied whether resilience buffered the association between stressful life events and delinquency. Other researchers (29) showed that that resilience weakened the impact of peer victimization on depression. None of these studies has established that resilience buffered the direct and indirect relations between stressful life events and adolescent NSSI. Guided by the experiential avoidance model of NSSI (22) and the protective factor model of resilience (23), the current research filled in these gaps by examining the mediating role of depression, and the moderating role of resilience, in the links between stressful life events and NSSI. The results supported our hypotheses concerning the environmental context in which NSSI occurs, and the role of individual differences in navigating that environment.

### Stressful life events and NSSI

The findings supported our hypothesis that adolescents who experienced stressful life events are more likely to engage in NSSI. This result confirmed the findings from previous studies that showed a significant positive correlation between stressful life events and adolescent NSSI (16–18, 44). This result also is consistent with what would be expected based on the general strain theory (15). These findings suggest that reducing stressful life events would be beneficial in reducing adolescent NSSI.

### The mediating role of depression

The findings supported our hypothesis that depression mediates the association between stressful life events and adolescent NSSI. Thus, depression appears to be one of the explanatory mechanisms that might explain why adolescents who experience stressful life events are more likely to engage in NSSI. These results are consistent with those from previous studies that found that stressful life events could increase the risk of depression (17, 21, 51, 52). Our results suggest that we should pay more attention to adolescents who have experienced stressful life events and teach them how to alleviate their depressive moods.

Our results also suggest that depression in response to stressful life events could in turn increase the risk of NSSI. According to the experiential avoidance model of NSSI (22), negative emotions usually precede NSSI, and adolescents may engage in NSSI as a means of avoiding these emotions. Our findings are consistent with this model and with other research showing that negative emotions (such as depression, anxiety, and anger expression) are a particularly significant trigger of adolescent NSSI (6, 16, 30, 44, 45, 53). Our results, together with those of earlier research, provide evidence of a mediated pathway in which stressful life events increase the risk of negative emotions, which in turn increases the risk of NSSI.

### The moderating role of resilience

Consistent with our hypothesis and with the protective factor model of resilience (23), we found that resilience moderated the mediation effect of depression in the relationship between stressful life events and NSSI. To be specific, high levels of resilience significantly weakened the impact of stressful life events on depression and on NSSI. These findings were in alignment with previous studies that found that resilience buffered the association between stressful life events and adolescents' negative outcomes (28–30).

According to the resilience theory (23), resilience may be a buffer against stress because it helps adolescents who experience stressful life events to mobilize their internal protective resources (such as coping skills and self-efficacy) and external protective resources (such as parental support and adult mentoring). In addition, adolescents with high levels of resilience have a positive outlook on life, and see adversity (such as stressful life events) as temporary (46). These characteristics should leave them less prone to depression and NSSI in the face of stressful life events.

### Limitations and future directions

First, this research was conducted using a cross sectional study design. Future studies can adopt a longitudinal design with multiple time points to further explore the bidirectional relationships between stressful life events, resilience, depression and NSSI. Second, because all data were based on adolescents' self-reports, the associations may be inflated due to shared method bias. Future research should use a variety of methods (such as interviews) and information sources (such as peers) to collect data. Third, this study only tested the mediating role of depression in the relationship between stressful life events and adolescent NSSI, and future research can test the mediating roles of other emotions such as anxiety (6), anger expression (16), and shame (47). Moreover, this study only explored one moderator, namely resilience, and future research can test whether other factors such as regulatory emotional self-efficacy (50) protect against the effects of stressful life events on adolescents' depression and NSSI. Fourth, the sample was recruited from one school in Central China, and the results need to be replicated in other Chinese and non-Chinese samples. Finally, Covid-related life events and stress, family status, and financial problems have not been assessed and need to be assessed in further studies.

### Implications for practice

The current research has two important implications for prevention and intervention. First, depression appears to be a mechanism linking stressful life events to adolescent NSSI, and reducing depression may help to prevent the influence of stressful life events on NSSI. For instance, schools can teach students to master positive and effective emotion regulation methods by starting mental health courses, which can help reduce depression in time. Second, the results showed that resilience was a protective factor that buffered against the effect of stressful life events. Interventionists can improve the level of adolescents' resilience by developing social skills, self-efficacy, and academic skills (23).

## Conclusions

The current study contributes to the relevant literature by highlighting the roles of depression and resilience in the relationship between stressful life events and NSSI among Chinese adolescents. Results showed that depression is a potential mechanism linking stressful life events to adolescent NSSI, and resilience may be a protective factor that buffers against this risk process. Specifically, the adverse impact of stressful live effects on NSSI through depression was weaker in adolescents with higher resilience. These findings can inspire practitioners to pay attention to the interaction of risk factors and protective factors when providing prevention and intervention for adolescent NSSI.

## Data availability statement

The raw data supporting the conclusions of this article will be made available by the authors, without undue reservation.

## Ethics statement

The studies involving human participants were reviewed and approved by the Ethics Committee of Hubei University of Science and Technology. Written informed consent to participate in this study was provided by the participants' legal guardian/next of kin.

## Author contributions

CW, ZL, and XJ conceived and designed the research. CW and QX collected and analyzed the data. CW, TM, QX, and CY reviewed and edited the manuscript. All authors contributed to the article and approved the submitted version.

## Funding

This study was supported by the Philosophy and Social Sciences Research Project of Hubei Provincial Department of Education (21Y211), the Hubei Educational Science Planning Project (2021GB109), and the Research Project of the Research Center for Rural Educational and Cultural Development, Key Research Base of Humanities and Social Sciences in Hubei Province (2021NJZD03 and 2018-19JZ15).

## Conflict of interest

The authors declare that the research was conducted in the absence of any commercial or financial relationships that could be construed as a potential conflict of interest.

## Publisher's note

All claims expressed in this article are solely those of the authors and do not necessarily represent those of their affiliated organizations, or those of the publisher, the editors and the reviewers. Any product that may be evaluated in this article, or claim that may be made by its manufacturer, is not guaranteed or endorsed by the publisher.
